# Supplementary material for: Modeling glioblastoma heterogeneity as a dynamic network of cell states
Source: Mol Syst Biol. 2021 Sep 16;17(9):e10105. doi: 10.15252/msb.202010105 (PMC8444284; doi:10.15252/msb.202010105)
Supplement: Supplementary file 5 — Source Data for Figure 3 [file MSB-17-e10105-s001.zip › Figure3A_sourcedata/GSEA_3065/nosigdb_state1.GseaPreranked.1622718409535/RICHARDS_NOWAKOWSKI_SCIENCE_2017_RG_EARLY_UPREG.html]

Details for gene set RICHARDS\_NOWAKOWSKI\_SCIENCE\_2017\_RG\_EARLY\_UPREG[GSEA]

|  || Dataset | state1 |
| Phenotype | NoPhenotypeAvailable |
| Upregulated in class | na\_pos |
| GeneSet | RICHARDS\_NOWAKOWSKI\_SCIENCE\_2017\_RG\_EARLY\_UPREG |
| Enrichment Score (ES) | 0.48662847 |
| Normalized Enrichment Score (NES) | 2.0733922 |
| Nominal p-value | 0.0 |
| FDR q-value | 0.0 |
| FWER p-Value | 0.0 |
Table: GSEA Results Summary

  

Fig 1: Enrichment plot: RICHARDS\_NOWAKOWSKI\_SCIENCE\_2017\_RG\_EARLY\_UPREG      
 Profile of the Running ES Score & Positions of GeneSet Members on the Rank Ordered List

  

| PROBE | GENE SYMBOL | GENE\_TITLE | RANK IN GENE LIST | RANK METRIC SCORE | RUNNING ES | CORE ENRICHMENT || 1 | ODC1 |  |  | 7 | 0.744 | 0.0115 | Yes |
| 2 | HMGA1 |  |  | 8 | 0.736 | 0.0237 | Yes |
| 3 | PKM |  |  | 14 | 0.676 | 0.0343 | Yes |
| 4 | PTTG1 |  |  | 40 | 0.520 | 0.0402 | Yes |
| 5 | TPI1 |  |  | 52 | 0.473 | 0.0469 | Yes |
| 6 | MYL6 |  |  | 68 | 0.420 | 0.0522 | Yes |
| 7 | RPS12 |  |  | 73 | 0.411 | 0.0586 | Yes |
| 8 | HINT1 |  |  | 78 | 0.402 | 0.0648 | Yes |
| 9 | PRDX6 |  |  | 79 | 0.398 | 0.0713 | Yes |
| 10 | LDHA |  |  | 80 | 0.394 | 0.0778 | Yes |
| 11 | PHLDA1 |  |  | 83 | 0.389 | 0.0840 | Yes |
| 12 | RPL8 |  |  | 92 | 0.381 | 0.0895 | Yes |
| 13 | ENO1 |  |  | 99 | 0.373 | 0.0950 | Yes |
| 14 | GAPDH |  |  | 121 | 0.346 | 0.0985 | Yes |
| 15 | CTNNAL1 |  |  | 123 | 0.343 | 0.1040 | Yes |
| 16 | RPS2 |  |  | 124 | 0.342 | 0.1097 | Yes |
| 17 | UBE2C |  |  | 125 | 0.340 | 0.1153 | Yes |
| 18 | RPL35 |  |  | 127 | 0.338 | 0.1208 | Yes |
| 19 | NPM1 |  |  | 129 | 0.335 | 0.1262 | Yes |
| 20 | TPM1 |  |  | 132 | 0.335 | 0.1315 | Yes |
| 21 | RPL12 |  |  | 137 | 0.329 | 0.1365 | Yes |
| 22 | RPS18 |  |  | 148 | 0.325 | 0.1408 | Yes |
| 23 | PPIA |  |  | 150 | 0.324 | 0.1460 | Yes |
| 24 | RPS6 |  |  | 151 | 0.321 | 0.1513 | Yes |
| 25 | H2AFZ |  |  | 153 | 0.321 | 0.1565 | Yes |
| 26 | LSM4 |  |  | 162 | 0.316 | 0.1609 | Yes |
| 27 | YBX3 |  |  | 163 | 0.314 | 0.1661 | Yes |
| 28 | HMGN2 |  |  | 164 | 0.313 | 0.1712 | Yes |
| 29 | SNRPB |  |  | 172 | 0.310 | 0.1756 | Yes |
| 30 | VIM |  |  | 175 | 0.308 | 0.1805 | Yes |
| 31 | CDKN3 |  |  | 178 | 0.307 | 0.1853 | Yes |
| 32 | RPS26 |  |  | 180 | 0.305 | 0.1902 | Yes |
| 33 | PGAM1 |  |  | 190 | 0.302 | 0.1943 | Yes |
| 34 | RPL41 |  |  | 191 | 0.301 | 0.1992 | Yes |
| 35 | RAN |  |  | 194 | 0.297 | 0.2039 | Yes |
| 36 | CKS2 |  |  | 195 | 0.297 | 0.2088 | Yes |
| 37 | RPS15 |  |  | 196 | 0.297 | 0.2137 | Yes |
| 38 | RPL18A |  |  | 198 | 0.296 | 0.2185 | Yes |
| 39 | BIRC5 |  |  | 202 | 0.295 | 0.2231 | Yes |
| 40 | CYCS |  |  | 203 | 0.294 | 0.2279 | Yes |
| 41 | RPS8 |  |  | 210 | 0.291 | 0.2321 | Yes |
| 42 | TPT1 |  |  | 220 | 0.287 | 0.2359 | Yes |
| 43 | RPS3 |  |  | 222 | 0.287 | 0.2405 | Yes |
| 44 | RPS21 |  |  | 233 | 0.283 | 0.2441 | Yes |
| 45 | GPX4 |  |  | 234 | 0.283 | 0.2488 | Yes |
| 46 | H3F3B |  |  | 249 | 0.277 | 0.2519 | Yes |
| 47 | SIVA1 |  |  | 252 | 0.276 | 0.2562 | Yes |
| 48 | RPL32 |  |  | 263 | 0.274 | 0.2597 | Yes |
| 49 | UQCRFS1 |  |  | 265 | 0.273 | 0.2641 | Yes |
| 50 | SLC25A3 |  |  | 275 | 0.269 | 0.2675 | Yes |
| 51 | GNG5 |  |  | 276 | 0.269 | 0.2720 | Yes |
| 52 | SNRPG |  |  | 287 | 0.264 | 0.2753 | Yes |
| 53 | RPL29 |  |  | 293 | 0.262 | 0.2791 | Yes |
| 54 | DEK |  |  | 296 | 0.261 | 0.2832 | Yes |
| 55 | RPL14 |  |  | 299 | 0.260 | 0.2872 | Yes |
| 56 | RPL7A |  |  | 311 | 0.257 | 0.2903 | Yes |
| 57 | PSMB7 |  |  | 312 | 0.257 | 0.2945 | Yes |
| 58 | RPL7 |  |  | 313 | 0.256 | 0.2988 | Yes |
| 59 | RPL19 |  |  | 314 | 0.256 | 0.3030 | Yes |
| 60 | NOL7 |  |  | 324 | 0.252 | 0.3062 | Yes |
| 61 | RPS7 |  |  | 331 | 0.250 | 0.3097 | Yes |
| 62 | POLR2L |  |  | 339 | 0.245 | 0.3130 | Yes |
| 63 | RPLP1 |  |  | 341 | 0.244 | 0.3169 | Yes |
| 64 | RPL24 |  |  | 344 | 0.243 | 0.3207 | Yes |
| 65 | CKS1B |  |  | 346 | 0.242 | 0.3246 | Yes |
| 66 | NDUFA6 |  |  | 347 | 0.242 | 0.3286 | Yes |
| 67 | RPS27A |  |  | 354 | 0.239 | 0.3319 | Yes |
| 68 | RPLP2 |  |  | 356 | 0.239 | 0.3357 | Yes |
| 69 | PTGES3 |  |  | 359 | 0.237 | 0.3394 | Yes |
| 70 | NOG |  |  | 381 | 0.231 | 0.3410 | Yes |
| 71 | RPL18 |  |  | 383 | 0.230 | 0.3447 | Yes |
| 72 | NACA |  |  | 391 | 0.228 | 0.3477 | Yes |
| 73 | HSPD1 |  |  | 397 | 0.227 | 0.3509 | Yes |
| 74 | RPS19 |  |  | 416 | 0.223 | 0.3527 | Yes |
| 75 | RPL9 |  |  | 426 | 0.222 | 0.3554 | Yes |
| 76 | RPS25 |  |  | 431 | 0.221 | 0.3586 | Yes |
| 77 | RPL13 |  |  | 434 | 0.220 | 0.3621 | Yes |
| 78 | RPS14 |  |  | 437 | 0.220 | 0.3655 | Yes |
| 79 | PSMB3 |  |  | 444 | 0.218 | 0.3684 | Yes |
| 80 | RPLP0 |  |  | 452 | 0.215 | 0.3712 | Yes |
| 81 | RPS23 |  |  | 453 | 0.215 | 0.3748 | Yes |
| 82 | CACYBP |  |  | 458 | 0.214 | 0.3779 | Yes |
| 83 | RBM8A |  |  | 464 | 0.213 | 0.3809 | Yes |
| 84 | TCP1 |  |  | 475 | 0.211 | 0.3833 | Yes |
| 85 | RPS3A |  |  | 476 | 0.211 | 0.3868 | Yes |
| 86 | RHEB |  |  | 491 | 0.207 | 0.3887 | Yes |
| 87 | PPA1 |  |  | 498 | 0.206 | 0.3915 | Yes |
| 88 | RPL30 |  |  | 500 | 0.206 | 0.3948 | Yes |
| 89 | RPL5 |  |  | 513 | 0.203 | 0.3968 | Yes |
| 90 | HSP90AB1 |  |  | 521 | 0.201 | 0.3994 | Yes |
| 91 | PSMA3 |  |  | 528 | 0.200 | 0.4021 | Yes |
| 92 | HSPB1 |  |  | 531 | 0.199 | 0.4051 | Yes |
| 93 | HSPE1 |  |  | 545 | 0.197 | 0.4070 | Yes |
| 94 | TPX2 |  |  | 556 | 0.194 | 0.4091 | Yes |
| 95 | PSMA1 |  |  | 563 | 0.192 | 0.4117 | Yes |
| 96 | BNIP3 |  |  | 570 | 0.190 | 0.4142 | Yes |
| 97 | RPL6 |  |  | 572 | 0.190 | 0.4172 | Yes |
| 98 | CCNB1 |  |  | 583 | 0.189 | 0.4193 | Yes |
| 99 | TAF9 |  |  | 596 | 0.186 | 0.4211 | Yes |
| 100 | TFAP2C |  |  | 602 | 0.186 | 0.4236 | Yes |
| 101 | G3BP1 |  |  | 611 | 0.184 | 0.4258 | Yes |
| 102 | RPL23A |  |  | 612 | 0.184 | 0.4288 | Yes |
| 103 | RPS28 |  |  | 620 | 0.183 | 0.4311 | Yes |
| 104 | FDFT1 |  |  | 635 | 0.181 | 0.4326 | Yes |
| 105 | SNRPD1 |  |  | 647 | 0.179 | 0.4344 | Yes |
| 106 | ANAPC16 |  |  | 653 | 0.178 | 0.4368 | Yes |
| 107 | RPL21 |  |  | 660 | 0.177 | 0.4391 | Yes |
| 108 | KIF5B |  |  | 678 | 0.175 | 0.4401 | Yes |
| 109 | SERF2 |  |  | 695 | 0.173 | 0.4413 | Yes |
| 110 | SNRPE |  |  | 707 | 0.171 | 0.4430 | Yes |
| 111 | RPS24 |  |  | 708 | 0.171 | 0.4458 | Yes |
| 112 | RPL39 |  |  | 718 | 0.170 | 0.4476 | Yes |
| 113 | RPL15 |  |  | 720 | 0.169 | 0.4503 | Yes |
| 114 | CNN3 |  |  | 727 | 0.168 | 0.4524 | Yes |
| 115 | CSNK2B |  |  | 733 | 0.167 | 0.4547 | Yes |
| 116 | POLR2J |  |  | 742 | 0.166 | 0.4566 | Yes |
| 117 | CENPN |  |  | 764 | 0.163 | 0.4570 | Yes |
| 118 | RPL10A |  |  | 769 | 0.162 | 0.4593 | Yes |
| 119 | SCAND1 |  |  | 786 | 0.159 | 0.4602 | Yes |
| 120 | SLIRP |  |  | 815 | 0.155 | 0.4598 | Yes |
| 121 | RPL23 |  |  | 840 | 0.153 | 0.4598 | Yes |
| 122 | GSTP1 |  |  | 851 | 0.151 | 0.4612 | Yes |
| 123 | GLO1 |  |  | 855 | 0.151 | 0.4634 | Yes |
| 124 | CCT4 |  |  | 858 | 0.150 | 0.4656 | Yes |
| 125 | HMGB3 |  |  | 907 | 0.144 | 0.4629 | Yes |
| 126 | CASP3 |  |  | 923 | 0.142 | 0.4637 | Yes |
| 127 | SVIP |  |  | 937 | 0.141 | 0.4646 | Yes |
| 128 | ANLN |  |  | 941 | 0.141 | 0.4666 | Yes |
| 129 | DPY30 |  |  | 964 | 0.138 | 0.4666 | Yes |
| 130 | CDCA3 |  |  | 994 | 0.134 | 0.4657 | Yes |
| 131 | HMGB1 |  |  | 995 | 0.134 | 0.4679 | Yes |
| 132 | WTAP |  |  | 1001 | 0.134 | 0.4696 | Yes |
| 133 | HMGN3 |  |  | 1008 | 0.133 | 0.4712 | Yes |
| 134 | SNRPA1 |  |  | 1032 | 0.130 | 0.4709 | Yes |
| 135 | MRPL47 |  |  | 1046 | 0.128 | 0.4716 | Yes |
| 136 | RPL22L1 |  |  | 1048 | 0.128 | 0.4736 | Yes |
| 137 | SRSF3 |  |  | 1076 | 0.125 | 0.4728 | Yes |
| 138 | BOC |  |  | 1100 | 0.122 | 0.4724 | Yes |
| 139 | ETF1 |  |  | 1121 | 0.120 | 0.4723 | Yes |
| 140 | TWISTNB |  |  | 1128 | 0.119 | 0.4736 | Yes |
| 141 | TOMM5 |  |  | 1133 | 0.119 | 0.4751 | Yes |
| 142 | TIPARP |  |  | 1142 | 0.118 | 0.4762 | Yes |
| 143 | OSER1 |  |  | 1207 | 0.112 | 0.4713 | Yes |
| 144 | CCNB2 |  |  | 1232 | 0.110 | 0.4706 | Yes |
| 145 | CCDC25 |  |  | 1239 | 0.109 | 0.4717 | Yes |
| 146 | EEF1D |  |  | 1244 | 0.109 | 0.4731 | Yes |
| 147 | CENPU |  |  | 1249 | 0.108 | 0.4744 | Yes |
| 148 | DDX39A |  |  | 1255 | 0.108 | 0.4757 | Yes |
| 149 | CAPZA1 |  |  | 1260 | 0.108 | 0.4770 | Yes |
| 150 | CENPW |  |  | 1278 | 0.106 | 0.4770 | Yes |
| 151 | FTH1 |  |  | 1296 | 0.104 | 0.4769 | Yes |
| 152 | KIF4A |  |  | 1303 | 0.103 | 0.4780 | Yes |
| 153 | MDK |  |  | 1323 | 0.101 | 0.4776 | Yes |
| 154 | ARRDC3 |  |  | 1347 | 0.099 | 0.4768 | Yes |
| 155 | CKAP2L |  |  | 1359 | 0.099 | 0.4773 | Yes |
| 156 | MRPL50 |  |  | 1364 | 0.098 | 0.4785 | Yes |
| 157 | CKB |  |  | 1368 | 0.098 | 0.4798 | Yes |
| 158 | TRIAP1 |  |  | 1374 | 0.098 | 0.4809 | Yes |
| 159 | RPL37 |  |  | 1385 | 0.096 | 0.4814 | Yes |
| 160 | CCDC59 |  |  | 1392 | 0.096 | 0.4823 | Yes |
| 161 | MAD2L1 |  |  | 1394 | 0.095 | 0.4838 | Yes |
| 162 | ATP6V0E1 |  |  | 1421 | 0.094 | 0.4826 | Yes |
| 163 | ERRFI1 |  |  | 1423 | 0.094 | 0.4840 | Yes |
| 164 | ACAA2 |  |  | 1425 | 0.094 | 0.4855 | Yes |
| 165 | UBE2T |  |  | 1440 | 0.093 | 0.4855 | Yes |
| 166 | SRP14 |  |  | 1445 | 0.093 | 0.4866 | Yes |
| 167 | RYBP |  |  | 1467 | 0.091 | 0.4859 | No |
| 168 | YWHAE |  |  | 1494 | 0.090 | 0.4846 | No |
| 169 | TYMS |  |  | 1501 | 0.089 | 0.4855 | No |
| 170 | RPF2 |  |  | 1542 | 0.086 | 0.4826 | No |
| 171 | CENPE |  |  | 1545 | 0.086 | 0.4839 | No |
| 172 | NDUFB1 |  |  | 1554 | 0.086 | 0.4844 | No |
| 173 | RPL13A |  |  | 1559 | 0.085 | 0.4854 | No |
| 174 | HMGB2 |  |  | 1570 | 0.085 | 0.4857 | No |
| 175 | HIST1H1E |  |  | 1608 | 0.082 | 0.4832 | No |
| 176 | UBB |  |  | 1620 | 0.081 | 0.4833 | No |
| 177 | KIF23 |  |  | 1638 | 0.080 | 0.4829 | No |
| 178 | SLC3A2 |  |  | 1646 | 0.079 | 0.4834 | No |
| 179 | AHSA1 |  |  | 1647 | 0.079 | 0.4847 | No |
| 180 | PBK |  |  | 1651 | 0.079 | 0.4857 | No |
| 181 | ANP32E |  |  | 1706 | 0.075 | 0.4812 | No |
| 182 | NAP1L1 |  |  | 1717 | 0.074 | 0.4814 | No |
| 183 | GPN1 |  |  | 1718 | 0.074 | 0.4826 | No |
| 184 | NUSAP1 |  |  | 1736 | 0.073 | 0.4820 | No |
| 185 | H1FX |  |  | 1752 | 0.072 | 0.4816 | No |
| 186 | PRC1 |  |  | 1794 | 0.070 | 0.4784 | No |
| 187 | FAM162A |  |  | 1805 | 0.069 | 0.4785 | No |
| 188 | CCNA2 |  |  | 1835 | 0.068 | 0.4765 | No |
| 189 | KIF18A |  |  | 1842 | 0.067 | 0.4770 | No |
| 190 | KIF22 |  |  | 1858 | 0.067 | 0.4765 | No |
| 191 | LEF1 |  |  | 1861 | 0.067 | 0.4774 | No |
| 192 | PBDC1 |  |  | 1870 | 0.066 | 0.4777 | No |
| 193 | USP1 |  |  | 1921 | 0.064 | 0.4734 | No |
| 194 | ZWINT |  |  | 1923 | 0.064 | 0.4744 | No |
| 195 | SFRP1 |  |  | 1944 | 0.063 | 0.4733 | No |
| 196 | USP39 |  |  | 1974 | 0.061 | 0.4712 | No |
| 197 | RACGAP1 |  |  | 1994 | 0.060 | 0.4702 | No |
| 198 | TUFT1 |  |  | 2002 | 0.060 | 0.4704 | No |
| 199 | DDIT3 |  |  | 2008 | 0.059 | 0.4709 | No |
| 200 | PSMD14 |  |  | 2025 | 0.058 | 0.4702 | No |
| 201 | DLGAP5 |  |  | 2028 | 0.058 | 0.4709 | No |
| 202 | HDAC9 |  |  | 2045 | 0.057 | 0.4701 | No |
| 203 | TBL1X |  |  | 2048 | 0.057 | 0.4709 | No |
| 204 | TYW3 |  |  | 2075 | 0.056 | 0.4691 | No |
| 205 | GMNN |  |  | 2097 | 0.055 | 0.4677 | No |
| 206 | PIN4 |  |  | 2099 | 0.055 | 0.4685 | No |
| 207 | RDH10 |  |  | 2113 | 0.054 | 0.4681 | No |
| 208 | ECT2 |  |  | 2150 | 0.053 | 0.4651 | No |
| 209 | NFKBIA |  |  | 2153 | 0.053 | 0.4658 | No |
| 210 | BET1 |  |  | 2160 | 0.053 | 0.4660 | No |
| 211 | PUF60 |  |  | 2176 | 0.052 | 0.4653 | No |
| 212 | RBBP8 |  |  | 2212 | 0.051 | 0.4624 | No |
| 213 | KIF2C |  |  | 2224 | 0.050 | 0.4621 | No |
| 214 | ZFAND2A |  |  | 2229 | 0.050 | 0.4625 | No |
| 215 | EIF3J |  |  | 2271 | 0.048 | 0.4589 | No |
| 216 | SMC4 |  |  | 2308 | 0.047 | 0.4559 | No |
| 217 | PRR11 |  |  | 2329 | 0.046 | 0.4545 | No |
| 218 | RNPS1 |  |  | 2347 | 0.045 | 0.4534 | No |
| 219 | HSPH1 |  |  | 2441 | 0.042 | 0.4443 | No |
| 220 | PTBP1 |  |  | 2448 | 0.041 | 0.4443 | No |
| 221 | DUSP10 |  |  | 2450 | 0.041 | 0.4449 | No |
| 222 | DPM1 |  |  | 2458 | 0.041 | 0.4448 | No |
| 223 | ZNF217 |  |  | 2460 | 0.041 | 0.4454 | No |
| 224 | COPS8 |  |  | 2487 | 0.040 | 0.4433 | No |
| 225 | ADAMTS9 |  |  | 2489 | 0.040 | 0.4439 | No |
| 226 | BTG3 |  |  | 2514 | 0.039 | 0.4420 | No |
| 227 | KNSTRN |  |  | 2515 | 0.039 | 0.4426 | No |
| 228 | ELL2 |  |  | 2543 | 0.038 | 0.4404 | No |
| 229 | HSP90AA1 |  |  | 2562 | 0.038 | 0.4391 | No |
| 230 | SPAG5 |  |  | 2566 | 0.038 | 0.4394 | No |
| 231 | NCAPG2 |  |  | 2704 | 0.033 | 0.4254 | No |
| 232 | HIST1H1C |  |  | 2712 | 0.033 | 0.4252 | No |
| 233 | RPS27L |  |  | 2723 | 0.032 | 0.4247 | No |
| 234 | DBF4 |  |  | 2751 | 0.031 | 0.4223 | No |
| 235 | MKI67 |  |  | 2760 | 0.031 | 0.4220 | No |
| 236 | CKAP2 |  |  | 2763 | 0.031 | 0.4223 | No |
| 237 | PPP4R2 |  |  | 2800 | 0.030 | 0.4190 | No |
| 238 | ARHGAP11A |  |  | 2814 | 0.030 | 0.4181 | No |
| 239 | PAPOLA |  |  | 2836 | 0.029 | 0.4164 | No |
| 240 | ATF3 |  |  | 2855 | 0.029 | 0.4149 | No |
| 241 | DUT |  |  | 3005 | 0.025 | 0.3995 | No |
| 242 | BTBD1 |  |  | 3064 | 0.023 | 0.3938 | No |
| 243 | TLK1 |  |  | 3089 | 0.023 | 0.3916 | No |
| 244 | DARS |  |  | 3142 | 0.022 | 0.3865 | No |
| 245 | PPP1CC |  |  | 3152 | 0.021 | 0.3859 | No |
| 246 | RAP2C |  |  | 3187 | 0.021 | 0.3826 | No |
| 247 | NOP58 |  |  | 3197 | 0.020 | 0.3820 | No |
| 248 | FXR1 |  |  | 3224 | 0.020 | 0.3795 | No |
| 249 | TAF7 |  |  | 3255 | 0.019 | 0.3767 | No |
| 250 | WDR43 |  |  | 3272 | 0.019 | 0.3753 | No |
| 251 | PPP1R15A |  |  | 3296 | 0.018 | 0.3732 | No |
| 252 | BICC1 |  |  | 3300 | 0.018 | 0.3731 | No |
| 253 | CDK1 |  |  | 3396 | 0.016 | 0.3633 | No |
| 254 | SAR1A |  |  | 3402 | 0.016 | 0.3631 | No |
| 255 | SPC24 |  |  | 3404 | 0.016 | 0.3632 | No |
| 256 | KCTD6 |  |  | 3441 | 0.015 | 0.3597 | No |
| 257 | CCDC18 |  |  | 3447 | 0.015 | 0.3594 | No |
| 258 | YAP1 |  |  | 3463 | 0.015 | 0.3580 | No |
| 259 | RSL24D1 |  |  | 3499 | 0.014 | 0.3545 | No |
| 260 | AJUBA |  |  | 3503 | 0.014 | 0.3544 | No |
| 261 | GPCPD1 |  |  | 3520 | 0.013 | 0.3530 | No |
| 262 | THUMPD1 |  |  | 3526 | 0.013 | 0.3527 | No |
| 263 | PCNA |  |  | 3540 | 0.013 | 0.3515 | No |
| 264 | WDR36 |  |  | 3556 | 0.013 | 0.3501 | No |
| 265 | HIST1H1D |  |  | 3604 | 0.012 | 0.3453 | No |
| 266 | H1F0 |  |  | 3617 | 0.011 | 0.3442 | No |
| 267 | CBWD2 |  |  | 3628 | 0.011 | 0.3434 | No |
| 268 | SP3 |  |  | 3699 | 0.010 | 0.3361 | No |
| 269 | TTK |  |  | 3724 | 0.009 | 0.3337 | No |
| 270 | CHMP1B |  |  | 3839 | 0.007 | 0.3218 | No |
| 271 | PER2 |  |  | 3867 | 0.007 | 0.3190 | No |
| 272 | IGF2BP2 |  |  | 4019 | 0.004 | 0.3031 | No |
| 273 | NFIL3 |  |  | 4056 | 0.003 | 0.2993 | No |
| 274 | EIF1AD |  |  | 4057 | 0.003 | 0.2994 | No |
| 275 | UHRF1 |  |  | 4092 | 0.003 | 0.2958 | No |
| 276 | ATF4 |  |  | 4139 | 0.002 | 0.2909 | No |
| 277 | IFT74 |  |  | 4143 | 0.002 | 0.2907 | No |
| 278 | ZNF433 |  |  | 4174 | 0.001 | 0.2875 | No |
| 279 | TCF3 |  |  | 4214 | 0.001 | 0.2834 | No |
| 280 | ALDH1B1 |  |  | 4217 | 0.000 | 0.2832 | No |
| 281 | NCAPG |  |  | 4261 | -0.000 | 0.2786 | No |
| 282 | HSPA1B |  |  | 4282 | -0.001 | 0.2765 | No |
| 283 | WBP4 |  |  | 4383 | -0.002 | 0.2659 | No |
| 284 | WEE1 |  |  | 4385 | -0.003 | 0.2659 | No |
| 285 | FOXN2 |  |  | 4398 | -0.003 | 0.2647 | No |
| 286 | NFYB |  |  | 4506 | -0.005 | 0.2534 | No |
| 287 | KIAA1217 |  |  | 4540 | -0.005 | 0.2500 | No |
| 288 | MCM3 |  |  | 4633 | -0.007 | 0.2403 | No |
| 289 | GLI2 |  |  | 4647 | -0.007 | 0.2391 | No |
| 290 | ZDHHC2 |  |  | 4692 | -0.007 | 0.2345 | No |
| 291 | CENPK |  |  | 4697 | -0.007 | 0.2342 | No |
| 292 | TCF7L2 |  |  | 4707 | -0.008 | 0.2334 | No |
| 293 | LDHB |  |  | 4712 | -0.008 | 0.2331 | No |
| 294 | SPC25 |  |  | 4740 | -0.008 | 0.2304 | No |
| 295 | MED30 |  |  | 4820 | -0.009 | 0.2221 | No |
| 296 | DEDD |  |  | 4933 | -0.012 | 0.2105 | No |
| 297 | MND1 |  |  | 4982 | -0.012 | 0.2056 | No |
| 298 | TIMELESS |  |  | 5058 | -0.013 | 0.1978 | No |
| 299 | HIST1H1A |  |  | 5105 | -0.014 | 0.1932 | No |
| 300 | HIST1H1B |  |  | 5125 | -0.014 | 0.1914 | No |
| 301 | TAF8 |  |  | 5151 | -0.015 | 0.1890 | No |
| 302 | KIF20B |  |  | 5169 | -0.015 | 0.1875 | No |
| 303 | BUB1B |  |  | 5187 | -0.015 | 0.1859 | No |
| 304 | DNAJB4 |  |  | 5195 | -0.015 | 0.1854 | No |
| 305 | PBX3 |  |  | 5220 | -0.016 | 0.1831 | No |
| 306 | IMMP1L |  |  | 5239 | -0.016 | 0.1815 | No |
| 307 | KIF14 |  |  | 5262 | -0.016 | 0.1794 | No |
| 308 | RSRC2 |  |  | 5272 | -0.016 | 0.1787 | No |
| 309 | ZNF134 |  |  | 5282 | -0.016 | 0.1781 | No |
| 310 | CHEK1 |  |  | 5330 | -0.017 | 0.1734 | No |
| 311 | ASNS |  |  | 5370 | -0.018 | 0.1695 | No |
| 312 | ZCCHC3 |  |  | 5440 | -0.019 | 0.1625 | No |
| 313 | TUBE1 |  |  | 5526 | -0.021 | 0.1539 | No |
| 314 | HAUS8 |  |  | 5537 | -0.021 | 0.1531 | No |
| 315 | HMGA2 |  |  | 5544 | -0.021 | 0.1528 | No |
| 316 | TP53 |  |  | 5557 | -0.021 | 0.1519 | No |
| 317 | EFNB1 |  |  | 5572 | -0.021 | 0.1508 | No |
| 318 | MIER1 |  |  | 5576 | -0.021 | 0.1508 | No |
| 319 | CHORDC1 |  |  | 5581 | -0.021 | 0.1508 | No |
| 320 | IER5 |  |  | 5645 | -0.023 | 0.1445 | No |
| 321 | GPBP1L1 |  |  | 5677 | -0.023 | 0.1415 | No |
| 322 | NSUN4 |  |  | 5688 | -0.023 | 0.1409 | No |
| 323 | ITGB3BP |  |  | 5717 | -0.024 | 0.1383 | No |
| 324 | PSIP1 |  |  | 5794 | -0.025 | 0.1307 | No |
| 325 | NUP50 |  |  | 5803 | -0.025 | 0.1302 | No |
| 326 | ZNF844 |  |  | 5821 | -0.026 | 0.1289 | No |
| 327 | NEK4 |  |  | 5861 | -0.026 | 0.1252 | No |
| 328 | RTKN2 |  |  | 5868 | -0.027 | 0.1250 | No |
| 329 | HSPA4L |  |  | 5938 | -0.028 | 0.1181 | No |
| 330 | MDM1 |  |  | 6073 | -0.030 | 0.1044 | No |
| 331 | RRM2 |  |  | 6214 | -0.033 | 0.0901 | No |
| 332 | NDC80 |  |  | 6240 | -0.034 | 0.0880 | No |
| 333 | SLC5A3 |  |  | 6314 | -0.035 | 0.0809 | No |
| 334 | RC3H2 |  |  | 6335 | -0.035 | 0.0793 | No |
| 335 | TGIF1 |  |  | 6360 | -0.036 | 0.0774 | No |
| 336 | CBFB |  |  | 6467 | -0.038 | 0.0668 | No |
| 337 | KIF11 |  |  | 6477 | -0.038 | 0.0664 | No |
| 338 | EEF1A1 |  |  | 6584 | -0.040 | 0.0559 | No |
| 339 | PPM1D |  |  | 6609 | -0.041 | 0.0540 | No |
| 340 | CMBL |  |  | 6644 | -0.042 | 0.0511 | No |
| 341 | PPIL4 |  |  | 6755 | -0.044 | 0.0401 | No |
| 342 | PEA15 |  |  | 6830 | -0.046 | 0.0330 | No |
| 343 | ZNF516 |  |  | 6867 | -0.046 | 0.0300 | No |
| 344 | CENPF |  |  | 6902 | -0.047 | 0.0272 | No |
| 345 | TOPORS |  |  | 6949 | -0.049 | 0.0231 | No |
| 346 | EAF1 |  |  | 6977 | -0.049 | 0.0210 | No |
| 347 | MYADM |  |  | 7175 | -0.055 | 0.0011 | No |
| 348 | SSB |  |  | 7242 | -0.056 | -0.0050 | No |
| 349 | NUP155 |  |  | 7251 | -0.057 | -0.0049 | No |
| 350 | EZH2 |  |  | 7260 | -0.057 | -0.0048 | No |
| 351 | REST |  |  | 7263 | -0.057 | -0.0041 | No |
| 352 | GSK3B |  |  | 7351 | -0.059 | -0.0123 | No |
| 353 | MTHFD2L |  |  | 7377 | -0.060 | -0.0140 | No |
| 354 | RNF139 |  |  | 7399 | -0.061 | -0.0152 | No |
| 355 | DUSP16 |  |  | 7416 | -0.061 | -0.0159 | No |
| 356 | PDCD4 |  |  | 7452 | -0.062 | -0.0186 | No |
| 357 | GLI3 |  |  | 7475 | -0.062 | -0.0199 | No |
| 358 | FANCD2 |  |  | 7483 | -0.063 | -0.0196 | No |
| 359 | EIF1B |  |  | 7516 | -0.064 | -0.0220 | No |
| 360 | CNTLN |  |  | 7518 | -0.064 | -0.0210 | No |
| 361 | ADNP |  |  | 7620 | -0.067 | -0.0306 | No |
| 362 | MCM4 |  |  | 7658 | -0.068 | -0.0334 | No |
| 363 | TMEM258 |  |  | 7671 | -0.069 | -0.0336 | No |
| 364 | TOP2A |  |  | 7678 | -0.069 | -0.0331 | No |
| 365 | KDM3A |  |  | 7808 | -0.074 | -0.0455 | No |
| 366 | ASPM |  |  | 7821 | -0.074 | -0.0456 | No |
| 367 | XPO1 |  |  | 7863 | -0.076 | -0.0487 | No |
| 368 | IRF2BP2 |  |  | 7887 | -0.077 | -0.0498 | No |
| 369 | SPCS1 |  |  | 7902 | -0.077 | -0.0500 | No |
| 370 | BARD1 |  |  | 7980 | -0.080 | -0.0569 | No |
| 371 | PNRC1 |  |  | 8006 | -0.081 | -0.0582 | No |
| 372 | CNIH1 |  |  | 8053 | -0.083 | -0.0617 | No |
| 373 | MSI1 |  |  | 8064 | -0.084 | -0.0614 | No |
| 374 | CEP152 |  |  | 8127 | -0.086 | -0.0665 | No |
| 375 | RBL1 |  |  | 8134 | -0.087 | -0.0657 | No |
| 376 | TMEM97 |  |  | 8161 | -0.088 | -0.0670 | No |
| 377 | DCLRE1C |  |  | 8164 | -0.088 | -0.0658 | No |
| 378 | INTS8 |  |  | 8209 | -0.090 | -0.0690 | No |
| 379 | RAD51AP1 |  |  | 8227 | -0.091 | -0.0693 | No |
| 380 | MIS18BP1 |  |  | 8274 | -0.093 | -0.0726 | No |
| 381 | CDC42EP4 |  |  | 8286 | -0.094 | -0.0722 | No |
| 382 | DYRK1A |  |  | 8330 | -0.096 | -0.0752 | No |
| 383 | TCF7L1 |  |  | 8342 | -0.096 | -0.0748 | No |
| 384 | TNFRSF19 |  |  | 8355 | -0.097 | -0.0745 | No |
| 385 | PIK3R3 |  |  | 8411 | -0.100 | -0.0787 | No |
| 386 | KIF15 |  |  | 8542 | -0.108 | -0.0907 | No |
| 387 | TRA2A |  |  | 8564 | -0.109 | -0.0911 | No |
| 388 | ARHGEF10 |  |  | 8608 | -0.112 | -0.0938 | No |
| 389 | CEP135 |  |  | 8623 | -0.113 | -0.0934 | No |
| 390 | GPC4 |  |  | 8624 | -0.113 | -0.0916 | No |
| 391 | MAGED2 |  |  | 8642 | -0.114 | -0.0915 | No |
| 392 | SMC2 |  |  | 8721 | -0.119 | -0.0978 | No |
| 393 | ZFP36L1 |  |  | 8789 | -0.124 | -0.1028 | No |
| 394 | PEG3 |  |  | 8808 | -0.125 | -0.1027 | No |
| 395 | HIST1H4C |  |  | 8820 | -0.126 | -0.1018 | No |
| 396 | ID4 |  |  | 8856 | -0.129 | -0.1034 | No |
| 397 | CREB3L2 |  |  | 8858 | -0.129 | -0.1013 | No |
| 398 | TMEM170A |  |  | 8873 | -0.131 | -0.1006 | No |
| 399 | DHX40 |  |  | 8906 | -0.134 | -0.1018 | No |
| 400 | SRPRB |  |  | 8910 | -0.135 | -0.0999 | No |
| 401 | ATAD2 |  |  | 8948 | -0.138 | -0.1016 | No |
| 402 | MAP1B |  |  | 8951 | -0.139 | -0.0995 | No |
| 403 | RBM39 |  |  | 8983 | -0.141 | -0.1004 | No |
| 404 | TSPAN6 |  |  | 8985 | -0.142 | -0.0982 | No |
| 405 | PTAR1 |  |  | 9056 | -0.148 | -0.1032 | No |
| 406 | SLC25A37 |  |  | 9067 | -0.149 | -0.1018 | No |
| 407 | AHI1 |  |  | 9077 | -0.150 | -0.1003 | No |
| 408 | TMSB15A |  |  | 9079 | -0.150 | -0.0979 | No |
| 409 | SALL1 |  |  | 9084 | -0.151 | -0.0958 | No |
| 410 | SREK1 |  |  | 9144 | -0.157 | -0.0995 | No |
| 411 | ZFP36L2 |  |  | 9162 | -0.160 | -0.0987 | No |
| 412 | HEY1 |  |  | 9215 | -0.168 | -0.1014 | No |
| 413 | LIFR |  |  | 9216 | -0.168 | -0.0986 | No |
| 414 | VEGFA |  |  | 9220 | -0.168 | -0.0962 | No |
| 415 | DNAJC1 |  |  | 9228 | -0.169 | -0.0941 | No |
| 416 | SCAF11 |  |  | 9232 | -0.170 | -0.0916 | No |
| 417 | HES1 |  |  | 9280 | -0.177 | -0.0937 | No |
| 418 | FAT1 |  |  | 9301 | -0.181 | -0.0928 | No |
| 419 | ZFHX4 |  |  | 9346 | -0.189 | -0.0944 | No |
| 420 | CCNL1 |  |  | 9347 | -0.189 | -0.0912 | No |
| 421 | ABHD3 |  |  | 9348 | -0.189 | -0.0881 | No |
| 422 | AGO3 |  |  | 9352 | -0.190 | -0.0853 | No |
| 423 | SYNE2 |  |  | 9362 | -0.193 | -0.0831 | No |
| 424 | HSP90B1 |  |  | 9365 | -0.193 | -0.0801 | No |
| 425 | HELLS |  |  | 9406 | -0.201 | -0.0810 | No |
| 426 | OFD1 |  |  | 9417 | -0.205 | -0.0787 | No |
| 427 | FKBP10 |  |  | 9455 | -0.215 | -0.0791 | No |
| 428 | PHLPP1 |  |  | 9463 | -0.217 | -0.0762 | No |
| 429 | MCAM |  |  | 9468 | -0.218 | -0.0731 | No |
| 430 | JAM3 |  |  | 9485 | -0.222 | -0.0711 | No |
| 431 | ZEB1 |  |  | 9492 | -0.224 | -0.0680 | No |
| 432 | PPP1R15B |  |  | 9494 | -0.224 | -0.0644 | No |
| 433 | NOTCH3 |  |  | 9501 | -0.226 | -0.0613 | No |
| 434 | ATP1B1 |  |  | 9542 | -0.236 | -0.0617 | No |
| 435 | TJP1 |  |  | 9592 | -0.257 | -0.0627 | No |
| 436 | BRCA1 |  |  | 9600 | -0.258 | -0.0591 | No |
| 437 | CREB5 |  |  | 9616 | -0.265 | -0.0564 | No |
| 438 | FGFR3 |  |  | 9621 | -0.266 | -0.0524 | No |
| 439 | RFX4 |  |  | 9655 | -0.285 | -0.0512 | No |
| 440 | SOX2 |  |  | 9680 | -0.298 | -0.0488 | No |
| 441 | SOX9 |  |  | 9754 | -0.358 | -0.0506 | No |
| 442 | ITGB8 |  |  | 9794 | -0.418 | -0.0479 | No |
| 443 | C1orf61 |  |  | 9813 | -0.479 | -0.0419 | No |
| 444 | PON2 |  |  | 9835 | -0.554 | -0.0350 | No |
| 445 | MDM4 |  |  | 9841 | -0.601 | -0.0256 | No |
| 446 | SEMA5A |  |  | 9860 | -0.749 | -0.0151 | No |
| 447 | TTYH1 |  |  | 9872 | -1.033 | 0.0007 | No |
Table: GSEA details [plain text format]

  

Fig 2: RICHARDS\_NOWAKOWSKI\_SCIENCE\_2017\_RG\_EARLY\_UPREG: Random ES distribution      
 Gene set null distribution of ES for **RICHARDS\_NOWAKOWSKI\_SCIENCE\_2017\_RG\_EARLY\_UPREG**
